# Supplementary material for: Identification and Characterization of Clostridium perfringens Atypical CPB2 Toxin in Cell Cultures and Field Samples Using Monoclonal Antibodies
Source: Toxins (Basel). 2022 Nov 17;14(11):796. doi: 10.3390/toxins14110796 (PMC9693285; doi:10.3390/toxins14110796)

Figure S1. Detection of atypical CPB2 in some field samples by PCR. F.: Faeces; I.C: Intestinal Content; R.S: Rectal Swab. PC: Positive Control (*C. perfringens* atypical *cpb2*+ strain); NC: Negative Control (*C. perfringens* *cpb2*- strain). Molecular ladder is reported.

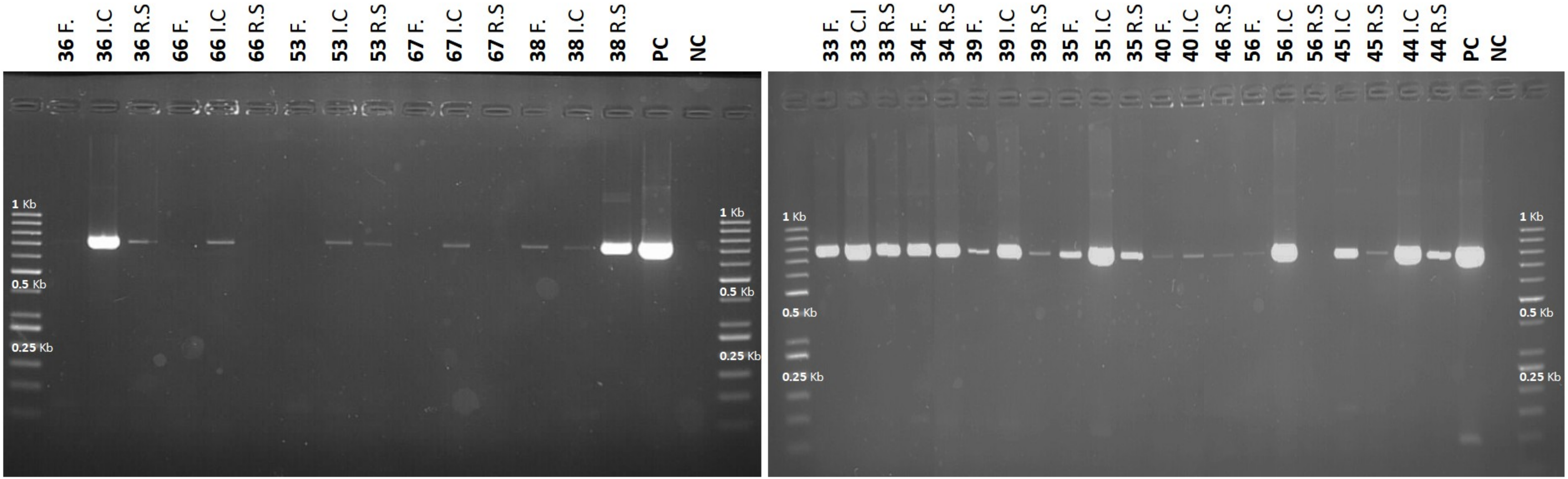

Supplement: Supplementary file 1 [file toxins-14-00796-s001.zip › Figure S1 final.pdf]
