# Supplementary material for: Identification and Characterization of Clostridium perfringens Atypical CPB2 Toxin in Cell Cultures and Field Samples Using Monoclonal Antibodies
Source: Toxins (Basel). 2022 Nov 17;14(11):796. doi: 10.3390/toxins14110796 (PMC9693285; doi:10.3390/toxins14110796)
Supplement: Supplementary file 1 [file toxins-14-00796-s001.zip › Table S1 final.pdf]

Table S1. Detection of atypical CPB2 on strain culture supernatants genotyped as atypical *cpb2*+. sELISA with 5C11E6, 2G3G6, 4E10E11 and conformation-dependent 23E6E6 Mabs. OD_{450} values are shown.

	MAbs			
Strain number	**5C11E6**	**2G3G6**	**4E10E11**	**23E6E6**
**C20**	0,1	0,096	0,12	0,1
**C21**	0,099	0,078	0,082	0,067
**C22**	0,06	0,062	0,054	0,054
**C23**	0,07	0,066	0,079	0,063
**C24**	0,052	0,07	0,052	0,061
**C25**	0,084	0,079	0,076	0,063
**C26**	0,048	0,059	0,049	0,086
