# Supplementary material for: Identification and Characterization of Clostridium perfringens Atypical CPB2 Toxin in Cell Cultures and Field Samples Using Monoclonal Antibodies
Source: Toxins (Basel). 2022 Nov 17;14(11):796. doi: 10.3390/toxins14110796 (PMC9693285; doi:10.3390/toxins14110796)
Supplement: Supplementary file 1 [file toxins-14-00796-s001.zip › Table S2 final.pdf]

Table S2. Detection of atypical CPB2 on strain culture supernatants genotyped as consensus *cpb2*+. sELISA with 5C11E6, 2G3G6, 4E10E11 and conformation-dependent 23E6E6 Mabs. OD_{450} values are shown.

	MAbs			
Strain number	**5C11E6**	**2G3G6**	**4E10E11**	**23E6E6**
**C27**	0,058	0,053	0,056	ND
**C28**	0,58	0,24	0,31	0,156
**C29**	0,062	0,064	0,055	0,055
**C30**	0,068	0,058	0,061	0,056
**C31**	0,072	0,076	0,073	0,064
**C32**	0,073	0,059	0,1	0,066
**C33**	0,059	0,104	0,071	0,066
**C34**	0,063	0,074	0,13	0,064
**C35**	0,058	0,093	0,074	0,07
**C36**	0,064	0,078	0,122	0,095
**C37**	0,061	0,11	0,083	0,058
**C38**	0,06	0,049	0,085	0,055
**C39**	0,058	0,051	0,1	0,053
**C40**	0,053	0,056	0,054	0,057
**C41**	0,05	0,056	0,06	0,063
**C42**	0,069	0,068	0,088	0,077
**C43**	0,057	0,056	0,055	0,057
**C44**	0,051	0,045	0,05	0,051
**C45**	0,047	0,058	0,057	0,056
**C46**	0,049	0,064	0,066	0,054
**C47**	0,055	0,054	0,075	0,07
**C48**	0,048	0,053	0,054	0,05
**C49**	0,047	0,047	0,05	0,057
**C50**	0,052	0,06	0,069	0,063
**C51**	0,054	0,066	0,075	0,07
**C52**	0,053	0,067	0,072	0,074
**C53**	0,054	0,053	0,062	0,065
