# Supplementary material for: Identification and Characterization of Clostridium perfringens Atypical CPB2 Toxin in Cell Cultures and Field Samples Using Monoclonal Antibodies
Source: Toxins (Basel). 2022 Nov 17;14(11):796. doi: 10.3390/toxins14110796 (PMC9693285; doi:10.3390/toxins14110796)
Supplement: Supplementary file 1 [file toxins-14-00796-s001.zip › Table S3 final.pdf]

Table S3. Detection of atypical CPB2 on strain culture supernatants genotyped as *cpb2*-. sELISA with 5C11E6, 2G3G6, 4E10E11 and conformation-dependent 23E6E6 Mabs. OD_{450} values are shown.

	MAbs			
Strain number	**5C11E6**	**2G3G6**	**4E10E11**	**23E6E6**
**C54**	0,057	0,065	0,11	0,064
**C55**	0,067	0,059	0,11	0,062
**C56**	0,067	0,062	0,107	0,069
**C57**	0,073	0,059	0,104	0,065
**C58**	0,071	0,061	0,106	0,062
**C59**	0,058	0,062	0,1	0,056
**C60**	0,056	0,054	0,108	0,062
**C61**	0,052	0,054	0,134	0,062
**C62**	0,051	0,052	0,12	0,055
**C63**	0,055	0,056	0,09	0,119
**C64**	0,148	0,067	0,065	0,065
**C65**	0,142	0,062	0,072	0,065
**C66**	0,75	0,49	0,297	0,1
