# Supplementary material for: Identification and Characterization of Clostridium perfringens Atypical CPB2 Toxin in Cell Cultures and Field Samples Using Monoclonal Antibodies
Source: Toxins (Basel). 2022 Nov 17;14(11):796. doi: 10.3390/toxins14110796 (PMC9693285; doi:10.3390/toxins14110796)
Supplement: Supplementary file 1 [file toxins-14-00796-s001.zip › Table S4 final.pdf]

Table S4. Detection of atypical CPB2 on field samples with both PCR and sELISA. OD_{450} values and PCR outcomes are shown. ND: not determined.

	**Faeces**		**Intestinal content**		**Rectal swab**	
Sample number	sELISA	PCR	sELISA	PCR	sELISA	PCR
**1**	0.084	Negative	0.087	Negative	0.071	Negative
**2**	0.067	Negative	0.093	Negative	0.06	Negative
**3**	0.086	Negative	0.07	Negative	0.063	Negative
**4**	0.058	Negative	0.053	Negative	0.053	Negative
**5**	ND	ND	0.061	Negative	ND	ND
**6**	0.063	Negative	0.077	Negative	0.067	Negative
**7**	0.057	Negative	0.056	Negative	0.05	Negative
**8**	0.05	Negative	0.051	Negative	0.048	Negative
**9**	0.05	Negative	0.05	Negative	0.05	Negative
**10**	0.049	Negative	0.052	Negative	0.051	Negative
**11**	0.079	Negative	0.069	Negative	0.064	Negative
**12**	0.076	Negative	0.065	Negative	0.06	Negative
**13**	0.08	Negative	0.107	Negative	0.071	Negative
**14**	ND	ND	0.075	Negative	0.061	Negative
**15**	0.089	Negative	0.054	Negative	0.08	Negative
**16**	0.05	Negative	0.071	Negative	0.056	Negative
**17**	ND	ND	0.093	Negative	0.094	Negative
**18**	0.054	Negative	0.053	Negative	0.049	Negative
**19**	0.071	Negative	0.073	Negative	0.086	Negative
**20**	0.053	Negative	0.076	Negative	0.05	Negative
**21**	0.053	Negative	0.05	Negative	0.058	Negative
**22**	0.051	Negative	0.075	Negative	0.051	Negative
**23**	0.063	Negative	0.069	Negative	0.054	Negative
**24**	ND	ND	0.078	Negative	ND	ND
**25**	0.08	Negative	0.055	Negative	0.131	Negative
**26**	0.306	Negative	0.062	Negative	0.059	Negative
**27**	0.28	Negative	0.05	Negative	0.124	Negative
**28**	ND	Negative	0.126	Negative	0.051	Negative
**29**	0.39	Negative	0.091	Negative	0.209	Negative
**30**	0.086	Negative	0.183	Negative	0.164	Negative
